# Boron improves cardiac contractility and fibrotic remodeling following myocardial infarction injury

**DOI:** 10.1038/s41598-020-73864-w

**Published:** 2020-10-13

**Authors:** Rihab Bouchareb, Michael Katz, Najla Saadallah, Yassine Sassi, Shakir Ali, Djamel Lebeche

**Affiliations:** 1 Cardiovascular Research Centre, The Icahn School of Medicine At Mount Sinai, New York, NY 10029 USA; 2 Diabetes, Obesity and Metabolism Institute, Department of Medicine, The Icahn School of Medicine At Mount Sinai, New York, NY 10029 USA; 3 Graduate School of Biological Sciences, The Icahn School of Medicine At Mount Sinai, New York, NY 10029 USA; 4 Department of Biochemistry, School of Chemical and Life Sciences, Jamia Hamdard (Deemed University), Hamdard Nagar, New Delhi, 110062 India

**Keywords:** Cardiology, Molecular medicine, Physiology, Cardiovascular biology

## Abstract

Myocardial fibrosis is a major determinant of clinical outcomes in heart failure (HF) patients. It is characterized by the emergence of myofibroblasts and early activation of pro-fibrotic signaling pathways before adverse ventricular remodeling and progression of HF. Boron has been reported in recent years to augment the innate immune system and cell proliferation, which play an important role in the repair and regeneration of the injured tissue. Currently, the effect of boron on cardiac contractility and remodeling is unknown. In this study, we investigated, for the first time, the effect of boron supplementation on cardiac function, myocardial fibrosis, apoptosis and regeneration in a rat model myocardial infarction (MI)-induced HF. MI was induced in animals and borax, a sodium salt of boron, was administered for 7 days, p.o., 21 days post-injury at a dose level of 4 mg/kg body weight. Transthoracic echocardiographic analysis showed a significant improvement in systolic and diastolic functions with boron treatment compared to saline control. In addition, boron administration showed a marked reduction in myocardial fibrosis and apoptosis in the injured hearts, highlighting a protective effect of boron in the ischemic heart. Interestingly, we observed a tenfold increase of nuclei in thin myocardial sections stained positive for the cell cycle marker Ki67 in the MI boron-treated rats compared to saline, indicative of increased cardiomyocyte cell cycle activity in MI hearts, highlighting its potential role in regeneration post-injury. We similarly observed increased Ki67 and BrdU staining in cultured fresh neonatal rat ventricular cardiomyocytes. Collectively, the results show that boron positively impacted MI-induced HF and attenuated cardiac fibrosis and apoptosis, two prominent features of HF. Importantly, boron has the potential to induce cardiomyocyte cell cycle entry and potentially cardiac tissue regeneration after injury. Boron might be beneficial as a supplement in MI and may be a good candidate substance for anti-fibrosis approach.

## Introduction

Myocardial fibrosis (MF) is associated with nearly all forms of heart diseases, particularly heart failure (HF)^1^. It is characterized by quantitative and qualitative changes in the extracellular matrix (ECM) in cardiac muscle in response to myocardial infarction injury, systemic diseases and other damaging insults affecting the heart^2^. Notably, MF is initially characterized by the transdifferentiation of fibroblasts and the emergence of myofibroblasts and early activation of pro-fibrotic signaling pathways before adverse ventricular remodeling^3^. The formation of the fibrous tissue is initially vital for preventing rupture of the ventricular wall and preserving myocardial structure but its persistence results in stiffening of the heart wall, and subsequently to diastolic dysfunction and arrhythmias and accelerates the progression to heart failure^4^. A better understanding of the process and finding innocuous substances capable of attenuating fibrosis may potentially improve the cardiac health.

Emerging evidences in cardiac research suggest the role proteins and signalling networks such as the β-catenin, GSK-3β and TGF-β1-SMAD-3 signalling networks as critical regulator of fibrosis, including the trans-differentiation of cardiac fibroblast to activated cardiac fibroblast or myofibroblast^1^. In the last couple of decades, the role of the innate immune system in chronic cardiomyopathy has been acknowledged with preclinical studies showing both positive and negative effects of the immune activation in heart failure. However, therapies directed against immune activation failed to show the beneficial effects of^5^. An augmented innate immune response in order to promote cardiac tissue repair and regeneration could be a better treatment option in heart failure or myocardial infarction (MI).

Boron is a metalloid present in soil and human food chain in trace amounts. It is a vital micronutrient in plants and plays an important role in animals, including the macrophage stimulation via the pattern recognition receptor ligand in murine model, and in in cellular processes such as apoptosis^6,7^. In human, an electrogenic Na^+^-coupled borate cotransporter, NaBC1, coded by *SLC4A11* gene has been identified^8,9^. Mutations in *SLC4A11* have been associated with endothelial dystrophy and a combined auditory and visual impairment, causing what is called the Harboyan syndrome^8,9^. In the literature, boron is reported to regulate a range of ECM proteins^10^, such as the expression of bone morphogenetic proteins (BMPs)^11,12^ and significantly improve the wound healing process^13^. In human, boron deficiency affects general health. Nutritional intake of boron lessens the adverse consequences of vitamin D deficiency (VDD)^14^; interestingly, VDD is a cardiovascular risk factor^15^. Boron, at low dose level, is also reported to impact (activate) the MAPK pathway and stimulate cell growth and proliferation^9^. It has been postulated that at the molecular level, boron may find novel usages in regenerative medicine^10^, restoring the structure and function of damaged tissues and organs. In view of the role of boron on macrophage polarization – an adaptive component of the innate immunity which plays an important and complex role throughout the acute inflammatory and regenerative response – and its effect on cell proliferation^6,7^, we hypothesized the potential beneficial effect of boron in cardiac tissue remodeling, cardiomyocyte cell cycle activity and regeneration post cardiac injury/MI. This is the first study of its kind where a metalloid (boron), a Group 13 chemical element of the Periodic Table, found in close proximity of carbon, one of the six elements (H, C, N, O, P and S) that came together to form life on earth, is reported to improve the cardiac health post-injury. The study implicates boron as a supplement/drug to attenuate fibrosis and for potential regeneration of the cardiac tissue post-injury.

## Results

### Boron improves cardiac function in adult post-myocardial infarction hearts

**Figure 1:**
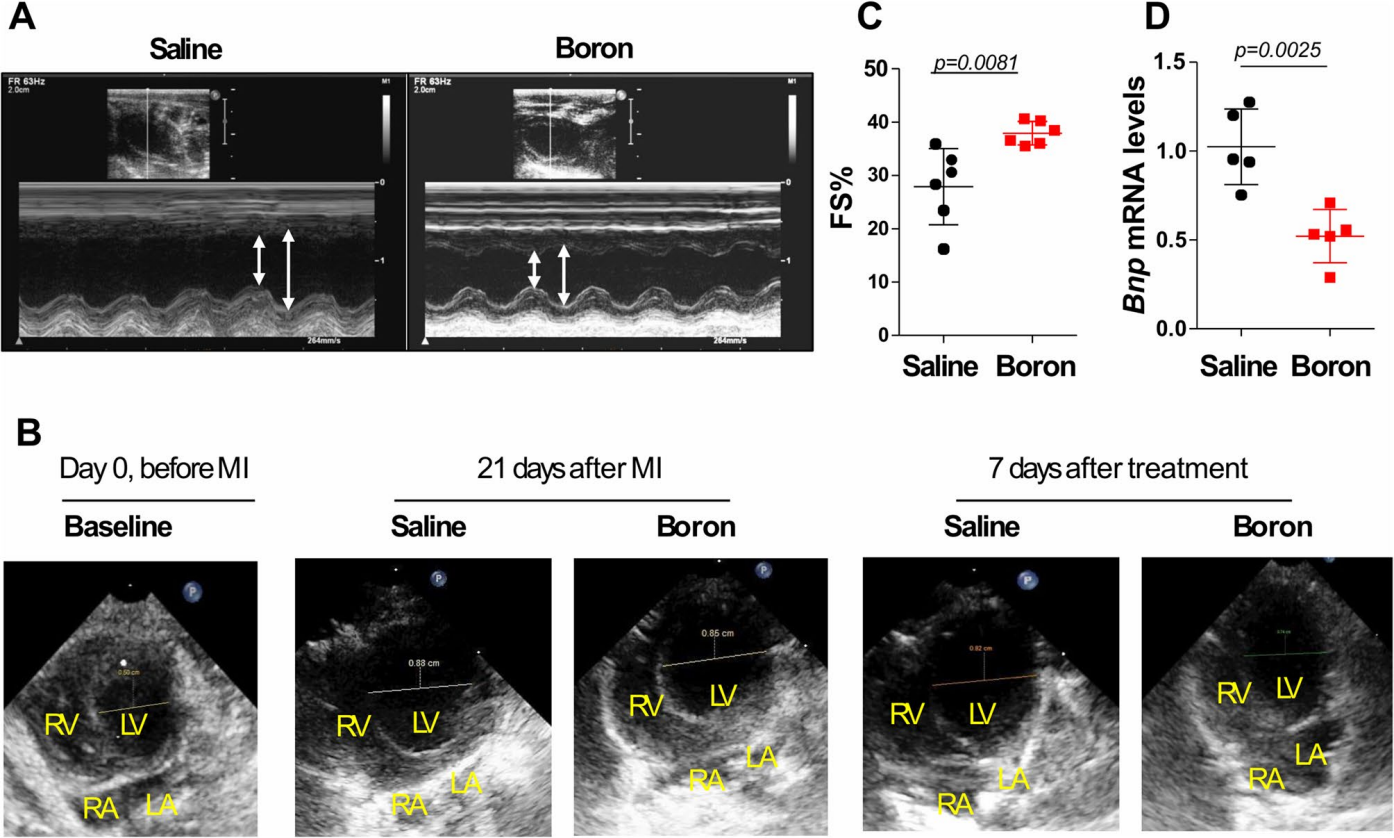
Echocardiographic analyses. (**A**) Representative raw tracings of rats M-mode echocardiography 1 week after saline or boron treatment. (**B**) Echocardiographic 4-chamber view showing the diameter of the left ventricle. *RV* right ventricle; *LV* left ventricle; *RA/LA* right and left atrium. Measurements indicate the diameter of the LV in 4 chamber view taken during diastole. (**C**) Echocardiographic measurements of LV fractional shortening (FS, %). D) qPCR of *Bnp* mRNA expression in saline and boron LV tissues, evaluated as fold change relative to the saline and normalized to 18S or HPRT rRNA endogenous control. n = 5–6; Values represent mean ± sd. Significance of differences as indicated.

We utilized two-dimensional echocardiography and M-mode echocardiography including conventional and tissue Doppler imaging to assess the effect of boron on cardiac function in boron-treated and saline control animals. Left ventricular (LV) function and dimensions were measured at baseline (Supplement Table 1), at 21 days after MI operation and 7 days post treatment (Table 1). Echocardiographic and cardiac morphometric analyses of saline- or boron-treated rats LV are shown in Table 2. Representative M-mode tracings and 4-chamber views of LV diameters are shown in Fig. 1A,B, respectively. M-mode analysis determined the saline and boron groups have significant modification of LVIDs dimensions (Table 1) and morphology (Fig. 1B) between baseline and follow up. The boron-treated rats also exhibited significantly increased contractility as assessed by the fractional shortening (FS; 27.88 ± 7.13% vs. 37.92 ± 2.19%, *P* = 0.0081) (Fig. 1C) and the ejection fraction (EF; 61.09 ± 11.83% vs. 75.54 ± 3.26%, *P* = 0.0043) (Table 2) compared to saline-treated group. The improvement in systolic function was associated with a significant reduction in the level of the heart failure marker, brain natriuretic peptide—*Bnp* following boron addition (Fig. 1D).

**Table 1:** Echocardiography analysis. Rats were subjected to MI for 21 days, then treated with NaCl (Saline) or Boron for 7 days. Echocardiographic analyses were performed before and after treatment with NaCl or Boron as indicated (n = 6).

	NaCl			Boron		
	Before	After	*P*	Before	After	*P*
BW (g)	370.08 ± 36.71	383.5 ± 30.66	0.44	389.3 ± 36.70	390.38 ± 37.03	0.25
IVSd (mm)	2.37 ± 0.61	2.24 ± 0.53	0.1	2.3 ± 0.61	2.38 ± 0.28	0.75
LVIDd (mm)	7.82 ± 1.02	8.26 ± 0.36	0.41	8.13 ± 0.67	7.71 ± 0.74	0.44
LVPWd (mm)	2.88 ± 0.53	3.25 ± 0.78	0.44	2.63 ± 0.53	2.75 ± 0.80	0.62
IVSs (mm)	3.11 ± 0.67	3.15 ± 0.81	0.56	2.72 ± 0.67	2.87 ± 0.72	0.84
LVIDs (mm)	5.43 ± 0.63	5.63 ± 0.89	0.9	5.57 ± 0.63	4.79 ± 0.58	0.031
LVPWs (mm)	3.264 ± 0.16	3.83 ± 0.41	0.031	2.93 ± 0.16	3.57 ± 0.64	0.026
EF(Teich) %	69.85 ± 7.70	61.09 ± 11.83	0.31	67.43 ± 7.70	75.54 ± 3.26	0.031
FS %	34.378 ± 5.55	27.88 ± 7.13	0.31	31.56 ± 5.55	37.92 ± 2.19	0.031
E′ (mm)	4.92 ± 0.56	5.09 ± 0.34	0.81	5.09 ± 0.56	5.64 ± 0.17	0.125
E (mm)	86.16 ± 15.79	82.92 ± 13.97	0.06	95.55 ± 15.79	83.49 ± 7.26	0.31
A (mm)	66.01 ± 20.69	64.49 ± 24.61	0.44	63.55 ± 20.69	70.25 ± 4.11	0.1
TAPSE	10.66 ± 1.37	11.25 ± 2.48	0.9	11.5 ± 1.38	16 ± 1.00	0.045
E/E′	17.68 ± 3.80	16.21 ± 1.83	0.13	19.03 ± 3.80	14.82 ± 1.45	0.125
E/A	1.35 ± 0.27	1.41 ± 0.41	0.63	1.56 ± 0.27	1.18 ± 0.09	0.04

*BW* body weight; *IVSd* interventricular septum thickness at end-diastole; *LVIDd* left ventricular internal dimension at end-diastole; *LVPWd*, left ventricular posterior wall thickness at end-diastole; *IVSs* Interventricular septum thickness at end-systole; *LVIDs* left ventricular internal dimension at end-systole; *LVPWs* left ventricular posterior wall thickness at end-systole; *EF* ejection fraction; *FS* fractional shortening; *TAPSE* tricuspid annular plane systolic excursion.

E wave, mitral inflow peak velocity in early diastole; A, peak velocity flow in late diastole; E/A ratio is the ratio of the early (E) to late (A) ventricular filling velocities; E′, early relaxation velocity on tissue; E/E′ ratio of transmitral flow to mitral annular velocity. Data shown as mean ± SD.

**Table 2:** Echocardiography analysis. Rats were subjected to MI for 21 days, then treated with NaCl (Saline) or Boron for 7 days. Echocardiographic analyses comparing heart function in Saline vs Boron groups as indicated (n = 6).

	Saline	Boron	*P*
BW (g)	383.5 ± 30.66	390.38 ± 37.03	0.33
IVSd (mm)	2.24 ± 0.53	2.38 ± 0.28	0.73
LVIDd (mm)	8.26 ± 0.36	7.71 ± 0.74	0.78
LVPWd (mm)	3.25 ± 0.78	2.75 ± 0.80	0.31
IVSs (mm)	3.15 ± 0.81	2.87 ± 0.72	0.51
LVIDs (mm)	5.63 ± 0.89	4.79 ± 0.58	0.086
LVPWs (mm)	3.83 ± 0.41	3.57 ± 0.64	0.51
EF(Teich) %	61.09 ± 11.83	75.54 ± 3.26	0.0043
FS %	27.88 ± 7.13	37.92 ± 2.19	0.0081
E′ (mm)	5.09 ± 0.34	5.64 ± 0.17	0.016
E (mm)	82.92 ± 13.97	83.49 ± 7.26	0.69
A (mm)	64.49 ± 24.61	70.25 ± 4.11	0.31
TAPSE	11.25 ± 2.48	16 ± 1.00	0.0079
E/E′	16.21 ± 1.83	14.82 ± 1.45	0.016
E/A	1.41 ± 0.41	1.18 ± 0.09	0.3

*BW* body weight; *IVSd* interventricular septum thickness at end-diastole; *LVIDd* left ventricular internal dimension at end-diastole; *LVPWd* left ventricular posterior wall thickness at end-diastole; *IVSs* interventricular septum thickness at end-systole; *LVIDs* left ventricular internal dimension at end-systole; *LVPWs* left ventricular posterior wall thickness at end-systole; *EF* ejection fraction; *FS* fractional shortening; *TAPSE* tricuspid annular plane systolic excursion.

E wave, mitral inflow peak velocity in early diastole; A, peak velocity flow in late diastole; E/A ratio is the ratio of the early (E) to late (A) ventricular filling velocities; E′, early relaxation velocity on tissue; E/E′ ratio of transmitral flow to mitral annular velocity. Data shown as mean ± SD.

**Figure 2:**
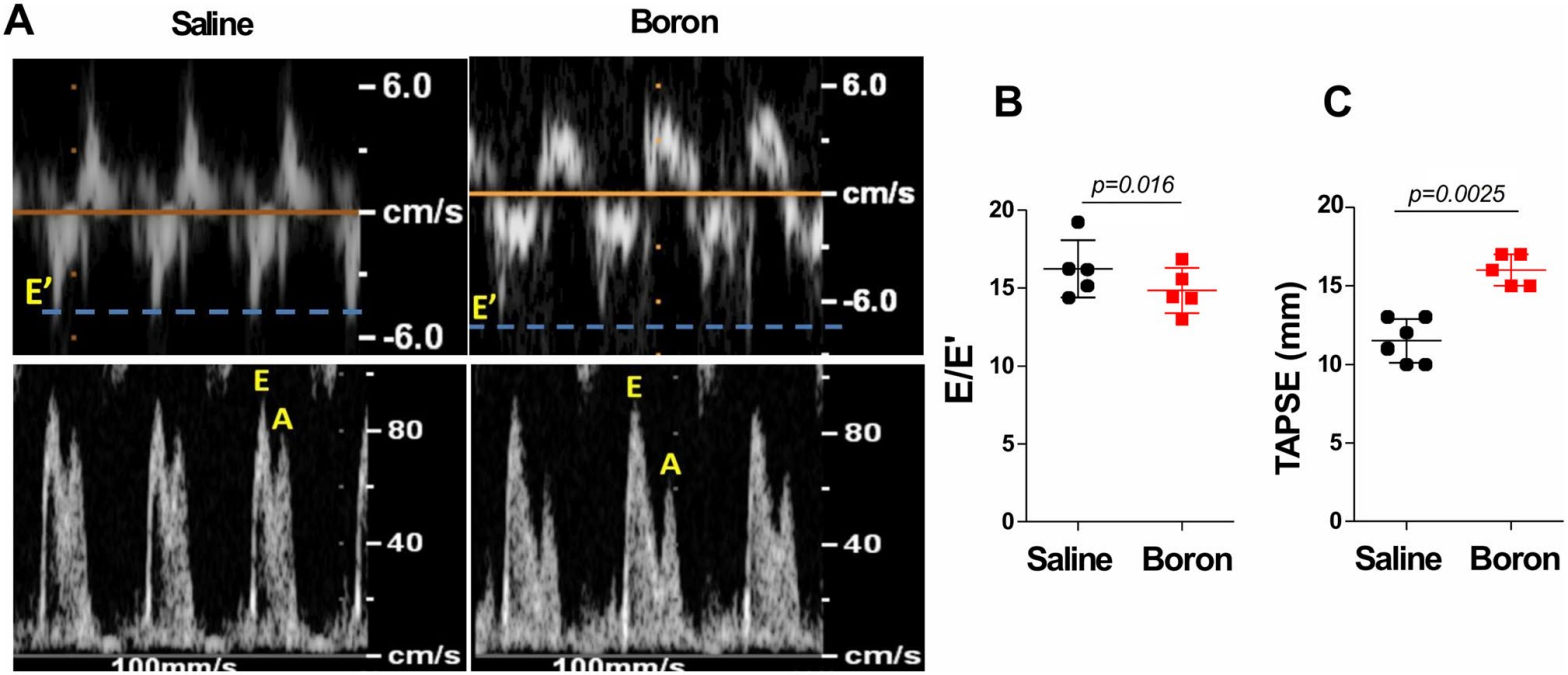
Tissue Doppler echocardiography. (**A**) Representative raw tracings of Tissue Doppler echocardiography of saline or boron-treated rats. (**B**) Measurements of Tissue Doppler-derived of the E/E′ ratio of transmitral flow to mitral annular velocity, an index of LV filling pressure. (**C**) Measurements of TAPSE, a parameter of RV systolic function which describes apex-to-base shortening. n = 5–6; Values represent mean ± sd. Significance of differences as indicated.

We further assessed diastolic function using Doppler echocardiography (Fig. 2A; Table 2). Mean E and late filling (A) ratio (E/A ratio) showed an abnormal pattern with a supernormal A wave in boron treated rats with no significant difference between the two groups (Fig. 2A); however, the boron-treated rats had significantly diminuend E/E′ ratio (16.21 ± 1.83 vs. 14.82 ± 1.45,  *P* = 0.016), in comparison with controls (Fig. 2B). The mitral E/E′ ratio on echocardiogram is an index of LV filling pressure and is an important indicator of LV diastolic function. Boron-induced reduction in E/E′ ratio is indicative of improved diastolic function. Furthermore, these results dovetail well with our findings regarding *Bnp* values (Fig. 1D), as studies have shown that the E/E´ ratio increases with the severity of heart failure and correlates with BNP values, and declines when heart failure improves.

Beside LV function, we also assessed right ventricular (RV) function using transthoracic echocardiography derived TAPSE (Tricuspid annular plane systolic excursion), a parameter of global RV function. Reduced values of TAPSE have been correlated with adverse outcome in heart failure^16–18^. Here we show that boron-treated rats had significantly elevated TAPSE (11.25 ± 2.48 vs. 16.00 ± 1.00,  *P* = 0.0079), in comparison with controls (Fig. 2C; Table 2), suggesting improvement in post-MI heart failure. Collectively, these findings document amelioration in global cardiac function with boron treatment following MI heart failure injury.

### Boron attenuates myocardial fibrosis (MF)

**Figure 3:**
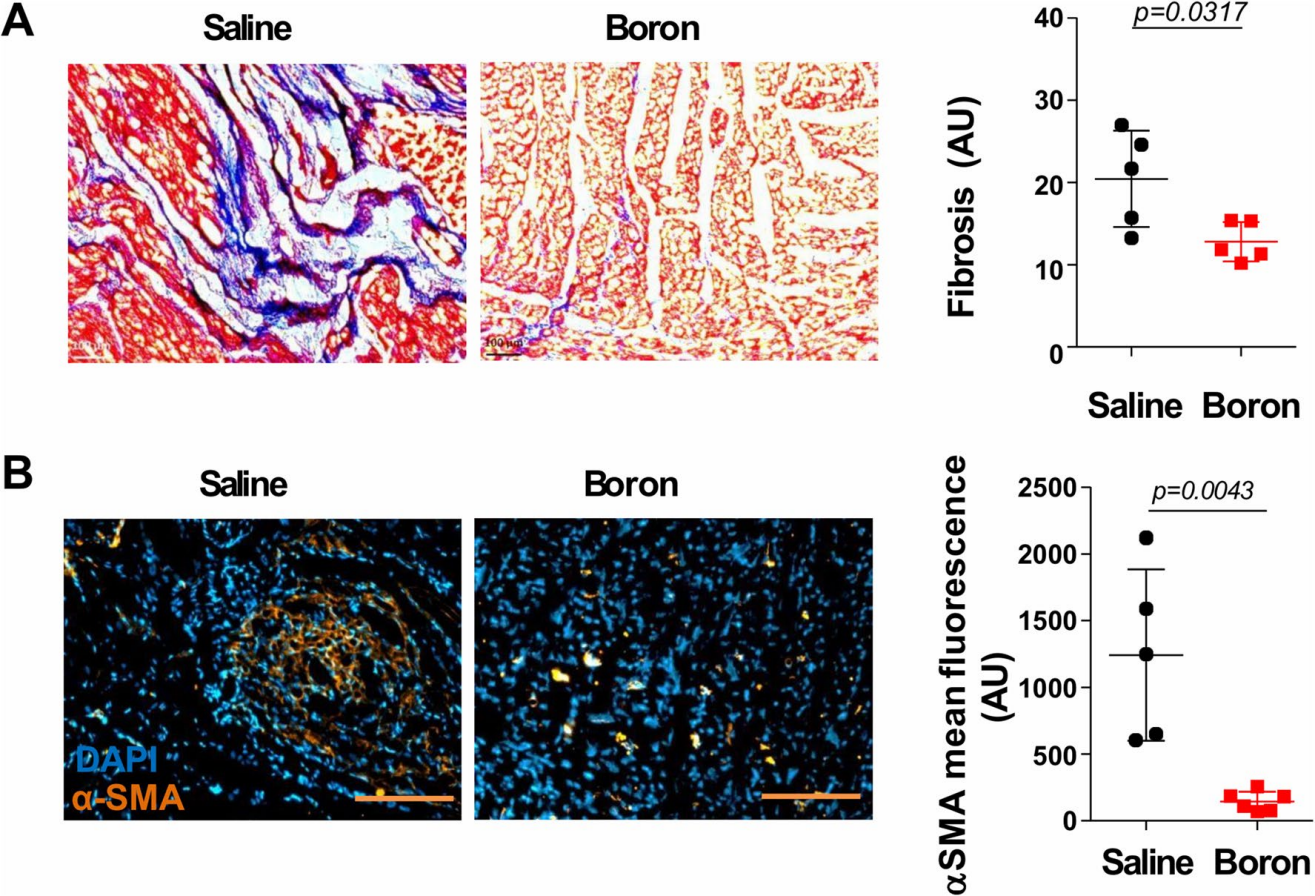
Histological assessment of myocardial fibrosis. (**A**) Representative micrographs of Masson Trichrome staining and quantification of fibrotic area in saline and boron-treated hearts. (**B**) Expression of αSMA determined by immunofluorescence staining and quantified in saline and boron-treated hearts. n = 5–6; Values represent mean ± sd. Significance of differences as indicated.

**Figure 4:**
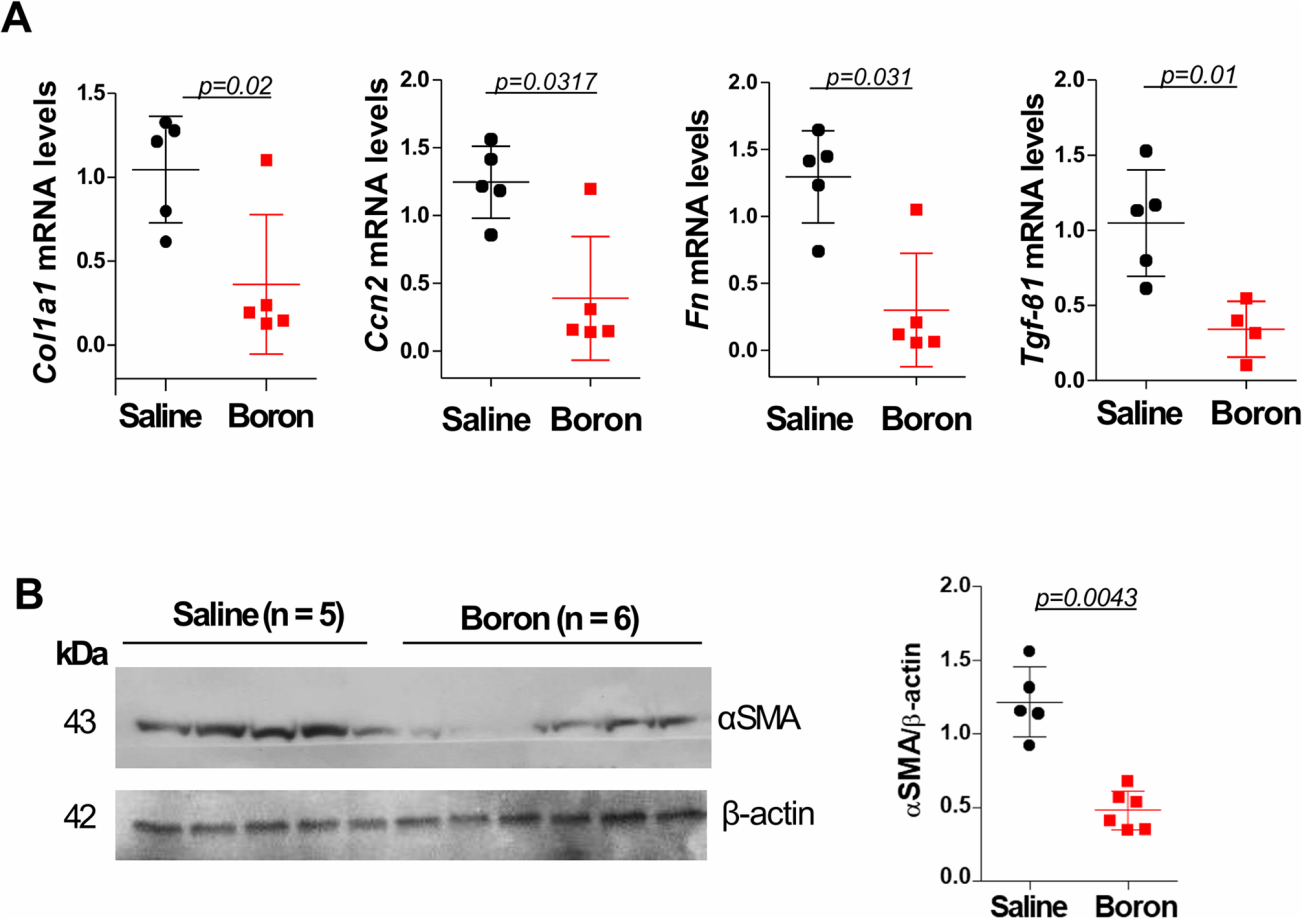
Molecular analysis of fibrosis markers. (**A**) The relative mRNA expression levels of Collagen 1a (*Col1a1*), cellular communication network factor 2 (*Ccn2*), fibronectin (*Fn*) and *Tgfβ1*, normalized to 18S or HPRT rRNA expression, in saline and boron-treated hearts. (**B**) Western blot analysis and densitometric quantification of αSMA expression. β-actin protein expression was used as loading control. n = 5–6; Values represent mean ± sd. Significance of differences as indicated.

Myocardial fibrosis and increased ECM turnover are hallmarks of cardiac hypertrophy and heart failure. The transdifferentiation of fibroblasts and increased deposition of ECM components results in myocardial stiffness and diastolic dysfunction. We therefore explored whether boron plays a role in the regulation of MF. Indeed, histological analysis of LV sections by Masson Trichrome staining and subsequent quantification of the fibrotic area showed less interstitial fibrosis in the boron-treated animals compared to saline controls (Fig. 3A). In addition, compared to saline, boron significantly reduced the expression of α-smooth muscle actin (αSMA) as determined by immunofluorescence staining (Fig. 3B) and western blot analysis (Fig. 4B), indicating reduced differentiation of fibroblasts. To elucidate the molecular changes of boron-induced fibrosis, we determined the expression levels of key profibrotic targets. mRNA analysis revealed that boron decreased the mRNA expression levels of collagen 1a (*Col1a1*), connective tissue growth factor (*Ctgf*)(also known as cellular communication network factor 2 (*Ccn2*)), fibronectin (*Fn*) and Tgf-β1 (Fig. 4A). These data clearly demonstrate that boron treatment is associated with decreased MF in vivo.

### Boron protects cardiomyocytes against apoptosis

**Figure 5:**
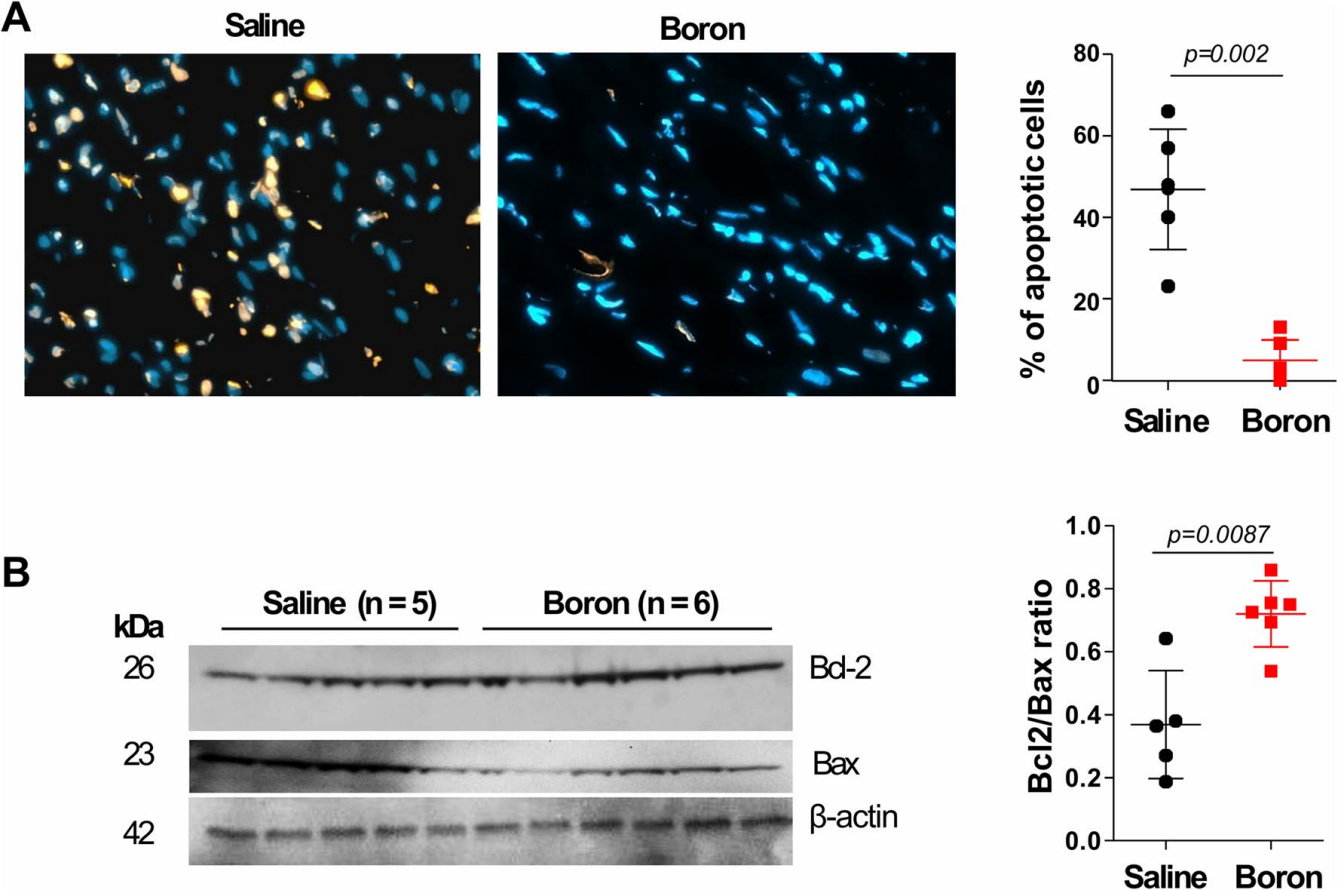
Assessment of apoptosis. (**A**) Representative epifluorescence images of TUNEL labeling and quantification of TUNEL-positive nuclei of LV histological sections from saline and boron-treated hearts. Arrows indicate TUNEL positive nuclei. Values are means ± sd measured from 10,000 nuclei. (**B**) Western blot analysis of Bcl-2 and Bax protein expression. β-actin protein expression was used as loading control. Densitometric analysis of the Bcl-2/Bax ratio is shown. Values represent mean ± sd, n = 5–6. Significance of differences as indicated. The samples in Fig. 5 derive from the same experiment as in Fig. 4 and that gels/blots were processed in parallel.

The decrease in MF displayed by boron prompted us to examine the rates of apoptosis in boron hearts. We found a significant decrease in the number of apoptotic cells in LV tissue sections from MI rats treated with boron compared to MI saline animals (4.833 ± 2.07% vs. 46.83 ± 6.02%; *P* = 0.002) as measured by Tunnel assay (Fig. 5A). The anti-apoptotic effect of boron was associated with higher protein levels of Bcl-2 (anti-apoptotic) and lower amounts of Bax (pro-apoptotic) in LV tissues from boron-treated animals (Fig. 5B). Consequently, the bcl-2/bax ratio, an important marker of myocardial cell survival probability, was significantly increased in the boron group when compared to saline-treated hearts (*P* = 0.0087). Altogether, these findings suggest that boron may induce a lower Bcl-2/Bax ratio that blocks the release of mitochondrial cell death signals and attenuates apoptosis.

### Boron stimulates cell cycle activity of cardiomyocytes in vivo and in vitro

**Figure 6:**
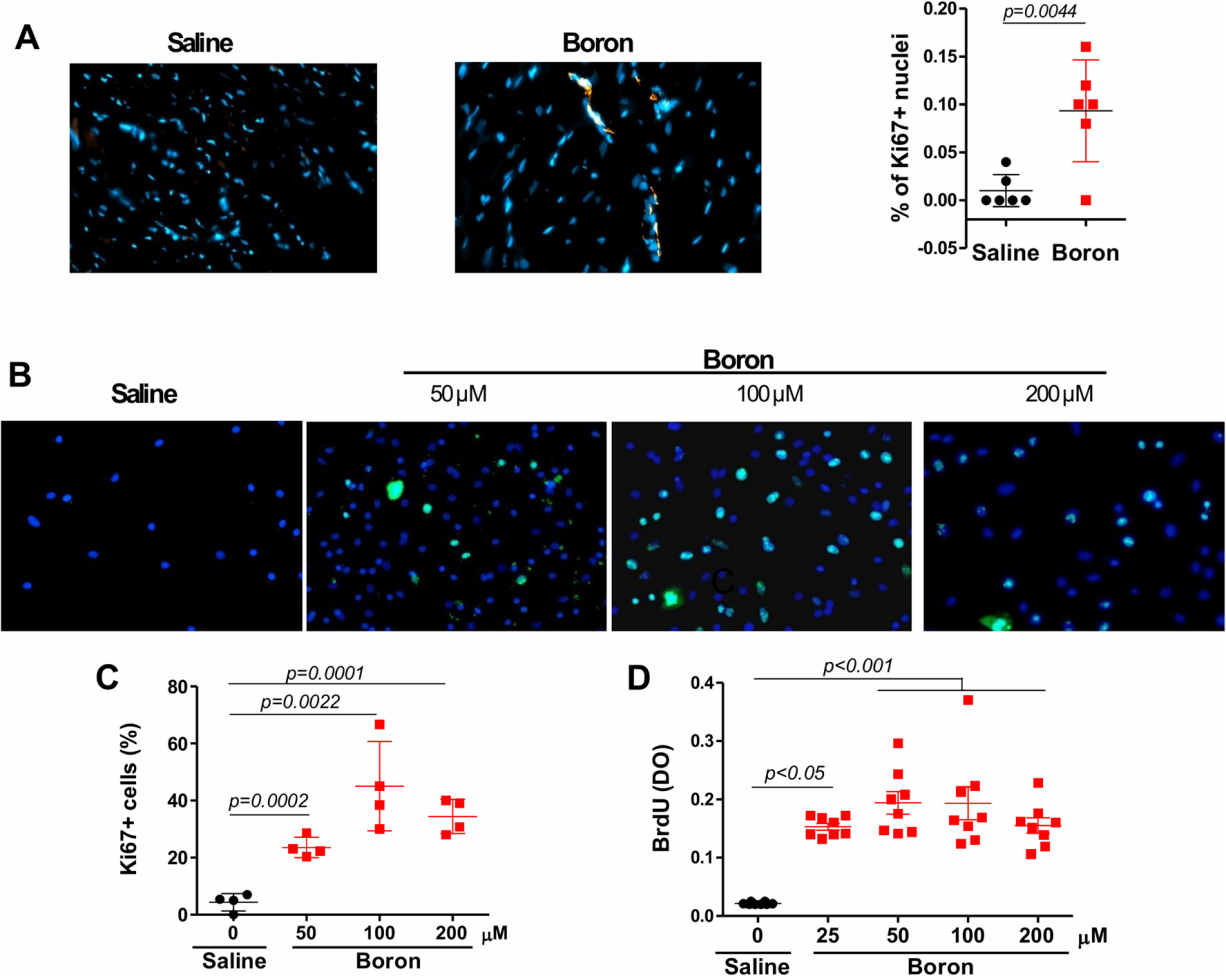
Effect of Boron on cardiac cell cycle activity. (**A**) Representative epifluorescence images of Ki67 labeling and quantification of Ki67-positive nuclei (%) of LV histological sections from saline and boron-treated hearts. Arrows indicate Ki67 positive nuclei. Values are means ± sd measured from 100 nuclei/section. (**B**) Ki67 staining and (**C**) quantitative analysis of Ki67-positive neonatal rat ventricular cardiomyocytes in (**B**) in the absence (saline) and presence of boron at the doses indicated. Values represent mean ± sd, % staining from at least 4 different experiments. (**D**) Quantitative analysis of BrdU-positive neonatal rat ventricular cardiomyocytes in the absence (saline) or presence of boron at the doses indicated. Values represent mean ± sd, n = 8; 100 cells/field. Significance of differences as indicated.

The improvement in MF and apoptosis prompted us to examine cell cycle entry and DNA synthesis in response to boron. Ki67 staining analysis indicated a higher Ki67 positive cell proportion in the boron-treated hearts compared to saline controls (0.010 ± 0.0068 vs. 0.093 ± 0.021; *P* = 0.0044) (Fig. 6A). This low but measurable rate of cardiomyocytes cycling is consistent with some reports of cardiomyocyte turnover in the heart^19,20^, but not others^21^. In addition, primary cardiomyocytes isolated from fresh heart tissues of neonatal rats also showed a dose-dependent increase in Ki67 positive myocytes in response to boron addition (Fig. 6B,C). These observations were further validated by nucleotide analog-incorporation assays by staining neonatal rat cardiomyocytes with BrdU. As seen in Fig. 6D, BrdU staining showed increased incorporation into cardiomyocytes in response to boron treatment compared to saline, suggesting enhanced entry into S phase of the cell cycle. These data indicate that boron directly modulates cardiomyocyte cell cycle activity in a cell autonomous manner, and may promote the endogenous regeneration in the adult heart.

## Discussion

Organisms respond to injury in order to restore morphological and functional integrity of the tissue and organ. Several non-mammalian vertebrates preserve the capacity for a scar-free healing and regeneration, but in human and most mammals, survival depends on the ability to repair and regenerate the tissue post-injury. In simple terms, the predictable response to any healing process comprises of haemostasis, inflammation, repair and remodelling, with inflammation serving as a preparatory process for the formation of new tissue post-injury^22^. A pathological repair results in accumulation of permanent fibrotic scar tissue at the site of injury and failure to return to normal^23^. Cardiac fibroblasts play a critical role in the repair and remodeling of the heart that occur following myocardial infarction because of their exceptional plasticity to undergo conversions between related but different cell types, such as myofibroblasts. In human heart, soon after an infarction insult, the myofibroblasts initially promote the formation of a protective fibrotic scar to prevent rupture of the wall. However, a persistent response results in chronic cardiac fibrosis and organ dysfunction^24,25^. Currently there is no FDA approved anti-fibrotic drug. In this study, we provide evidence that boron induces cardiomyocyte cell cycle activity and improves cardiac function and fibrosis post-MI injury. It is the first study to report on the beneficial effects of boron in heart failure.

We observed a substantial improvement in cardiac function upon treatment with boron, as judged by positive changes in both systolic and diastolic parameters. Boron-induced amelioration in systolic function in our study was documented by significant modification in LV and RV contractility following MI heart failure injury. LV diastolic function was also improved with boron as documented by amelioration in the mitral E/E′ ratio, an index of LV filling pressure. Studies have shown that the E/E´ ratio increases with the severity of heart failure and correlates with values of the heart failure marker, BNP, and declines when heart failure improves. Indeed we observed in our study that the improvement in systolic and diastolic function was associated with a significant reduction in the level of *Bnp* mRNA following boron addition, further highlighting boron’s cardiac beneficial outcomes.

The global improvement in heart function was associated with reduction in myocardial fibrosis and apoptosis, two hallmarks of cardiac hypertrophy and heart failure; conceivably suggesting that attenuation of fibrosis and apoptosis following boron treatment contribute to preservation of myocardial tissue and subsequently lead to cardiac function improvement. Decreased fibrosis and apoptosis in boron-treated hearts, which coincided with reduced expression of fibrotic apoptotic molecular markers, clearly establish boron as an anti-fibrotic mediator and a positive regulator of cardiac function. Nevertheless, gaining further mechanistic insights into boron elicited cardiac protection would be instrumental in further improving its action.

In our study, we observed a clear-cut effect of boron on cell cycle entry and DNA synthesis of cardiomyocytes, highlighting its potential role in cardiac regeneration post-injury. Earlier, boron has been indicated for its role in bone regeneration, in the form of Mesoporous bioactive glass scaffolds, where boron incorporation significantly enhanced osteoblast proliferation and Collagen I and Runx2 expressions, significantly contributing in bone tissue engineering^26^. The presence of a borate transporter (NaBC1) in cells indicates the importance of maintaining a steady state of boron in the cytoplasm. An increase in cardiomyocyte cell cycle activity observed in this study post-MI may be partly attributed to the stabilizing effect of boron on mitochondrial membrane potential, as we reported previously^7^, thus promoting conditions that favour cell survival and theoretically cardiomyocyte proliferation. In fact, a reliable selective differentiation method of stem cells into functional cardiomyocytes is a prerequisite to succeed in the attempts to transplant cells^27^, where boron may assist.

In conclusion, myocardial fibrosis is a major pathophysiological mechanism in heart failure where existing therapies are insufficient. Nearly every form of the heart disease is associated with myocardial fibrosis. In this study, we have reported an inhibitory role of boron on myocardial fibrosis and to promote regeneration and repair. The beneficial effects of boron on cardiac fibrosis and overall cardiac health post-injury epitomize a potential intervention strategy in the resolution of cardiac tissue injury. A better understanding of the exact spatiotemporal description of the elements of the inflammatory and fibrotic pathways with regard to boron might lead to a novel strategy in the management and treatment of heart failure.

## Limitations

We acknowledge there are some limitations to our study. We used somewhat small numbers of rats and the duration of boron treatment was also for a short period, 1 week. This may explain the lack of noticeable modification in LV mass observed. It is quite possible that longer treatment periods are required to cause evident structural and morphological changes given the complexity of the in vivo physiological settings. However, the clinical relevance of our findings deserves further investigation. It would be interesting to evaluate the potential effects of boron in subjects with early stage heart failure with preserved ejection fraction (i.e. 3–7 days post MI), when the structural changes in the heart and vasculature are still reversible rather than in later stages which are associated with advanced myocardial structural remodeling and fibrosis. Furthermore, we have not explicitly investigated the molecular mechanisms underlying boron beneficial effects on cardiac function and cardiac regeneration post-MI injury. However, in view of our recent publication—suggesting boron to stimulate the synthesis and secretion of chemical mediators of inflammation (CMI) (such as TNF-α, IL-1β, IL-6 and NO) via TLR pathway and the effect of boron on macrophage polarization (an adaptive component of the innate immunity which plays an important and complex role throughout the acute inflammatory and regenerative response) and its effect on cell proliferation^6,7^, it is conceivable to propose M1 polarization as an important mechanism of action of boron, whereby CMI produced by the M1 macrophage may provide a novel therapeutic approach in myocardial infarction by promoting tissue regeneration post-injury.

## Methods and materials

### Myocardial infarction

Animals were handled as approved by the Mount Sinai Institutional Animal Care and Use Committee in accordance with the Guidelines for the Care and Use of Laboratory Animals published by the National Institutes of Health. Sprague Dawley rats (180–200 g) underwent left anterior descending (LAD) ligation as previously described^28^.

### Boron in vivo delivery protocol and tissue harvest

Three weeks post myocardial infarction (day 21), rats with evident hyperdynamic left ventricle (LV) by echocardiography were randomly chosen to receive saline injection or borax, a sodium salt of boron, at a daily dose of 4 mg/kg body weight for 7 days. (n = 6/group). At end of study (day 30), echocardiographic measurements were performed and animals were anaesthetized with 5% isoflurane and then killed by cervical dislocation; heart tissues were collected and a ring of left ventricular (LV) tissue was embedded for histological studies and the rest of the LV tissue was frozen for RNA and protein analysis.

### Echocardiographic analysis

Transthoracic echocardiography was performed using a vivid 7 echocardiography apparatus with a 14 MHz probe (i13L probe, Philips Healthcare) as we described previously^29–31^. Doppler echocardiography was performed with S12 probe to visualize the 4-chamber view. Animals were sedated with ketamine 80–100 mg/kg injected intraperitoneally. Long axis parasternal views and short axis parasternal two dimensional (2D) views, at the mid-papillary level, of the LV were obtained. The analyses were performed offline blinded to treatment group and to study time point using a workstation equipped with the Echopac PC software (GE Vingmed Ultrasound; Horten, Norway). All measurements were averaged over six cardiac cycles. M mode images were obtained by 2D guidance from the parasternal short axis view for the measurements of LV wall thickness of the septum (IVSd) and the posterior wall (LVPWd), LV end diastolic diameter (LVIDd) and LV end systolic diameter (LVIDs) as well as to calculate the LV fractional shortening and ejection fraction (FS, %). EF was calculated from M mode images and from the 4-chamber view in diastole and systole by using Simpson method as it was described previously^32^.

In order to evaluate LV diastolic function, mitral inflow was obtained using pulsed-wave Doppler from apical four-chamber view^30^.

### Quantitative real-time RT-PCR gene expression analysis

Relative gene expression was determined using two-step quantitative qRT-PCR. Quantitative PCR reactions were performed with Power SYBR Green Master Mix (Applied Biosystems, Forest City, CA) on an ABI Prism 7500 Real Time PCR System. Fold changes were calculated using the ΔΔCt method with normalization to 18S or HPRT rRNA endogenous control.

### Western blotting analysis

Protein expression was evaluated in LV lysates by Western blot analysis according to standard procedures. Protein lysates were matched for protein concentration, separated by SDS-PAGE and transferred onto PVDF membranes (Bio-Rad) that were incubated with appropriate primary antibodies: αSMA (Abcam) and bcl2, Bax (Cell Signaling Technology). Densities of the immunoreactive bands from 5 to 6 rat hearts were evaluated using NIH ImageJ. The samples used in the immunoblotting analyses derive from the same experiments and that gels/blots were processed in parallel. Protein loading was verified against β-actin (Sigma-Aldrich) densities.

### Histological assessments and fluorescent immunostaining

#### Assessment of fibrosis

Serial 8 µm frozen sections from LV tissue were stained with Masson trichrome to quantify cardiac fibrosis following the manufacturer’s instructions (Sigma). Using a Zeiss Axiocam microscopy in 20 × magnification field, slides were measured under transmitted light . The extent of fibrosis in myocardial tissue sections was quantified in the entire cross-sectional area of ventricular myocardium with ImageJ software as the relative area of positive Masson trichrome stained area (blue fibrosis) normalized to the total tissue area in boron and saline control animals, as we previously described^31,33^.

#### Assessment of apoptosis

Serial 8 µm frozen sections from LV tissue were fixed in 1% paraformaldehyde for 10 min at room temperature and post-fixed in Ethanol/Acetic acid (2:1) for 5 min at 20 °C. Detection of apoptotic cells was achieved by direct immuno-fluorescence detection using terminal deoxynucleotidyl transferase (TdT)-mediated dUTP nick end labeling (TUNEL) (ApopTag detection kit, Millipore), as we previously described^31,33^. Tissue sections were then counter-stained with DAPI stain (Victor), and viewed with a micro-optics microscope (Carl Zeiss) equipped with filter sets for rhodamine and DAPI staining. To quantify apoptosis, six to seven randomly selected microscopic fields per section zone of each ventricular sample were examined. The percentage of apoptotic cells was determined by counting the total number of nuclei and TUNEL positive nuclei (apoptotic cells). Sections of interest were photographed using a microscope-integrated 35-mm camera.

#### Immunohistochemistry of heart tissues

Serial 8 µm frozen sections from LV tissue were fixed in 1% paraformaldehyde for 10 min at room temperature and post-fixed in Ethanol/Acetic acid (2:1) for 5 min at 20 °C. Sections were blocked with PBS BSA 1% for 1 h before immunolabeling with primary antibodies against: α-SMA (Abcam), and Ki67 (anti-Ki67; ThermoFischer Scientific). A fluorescent secondary antibody tagged alexa-red-555 was used against the primary antibody. Stained sections were scanned using an epifluorescence Zeiss Axiocam microscope equipped with a digital camera. The α-SMA + and Ki67 + mean fluorescence was calculated with NIH ImageJ software (NIH) in each section, comparing it with the size of the whole area.

### Isolation and culture of neonatal rat ventricular myocytes

Neonatal rat ventricular myocytes (NRVM) were isolated by enzymatic dissociation of cardiac ventricle from 1 to 2 days old Sprague–Dawley pups using the neonatal cardiomyocyte isolation system according to the manufacturer’s instructions (Worthington, Lakewood, NJ).

### Cardiomyocytes cell cycle entry and DNA synthesis assays

NRVM were seed into 96-well plate at a density of 1 × 10^4 cells/well. Cells were starved for 36 h (0.1% FBS) and cultured for an additional 48 h in the presence of (saline or presence of different doses of boron (50–200 µM) as indicated on the figures. In the last 24 h 30 μM BrdU was added to measure incorporation of BrdU during DNA synthesis, following manufactures’ instructions (Roche). Blank samples without cells were analyzed to subtract absorbance resulting from non-specific binding of BrdU and anti-BrdU antibody to the surface of microplate. Another batch of cells was treated in the same way and stained with Ki67 antibodies (Thermo Fischer Scientific). Nuclei were visualized with DAPI (4′,6′-diamidino-2-phenylindole, 0.5 μg/mL, Sigma). Staining was assessed using NIH ImageJ software (NIH).

### Statistical analysis

Statistical significance was analyzed with Student's paired and unpaired t test, followed by Bonferroni's method for post hoc pair-wise multiple comparisons. *P* < 0.05 was considered significant.

## Supplementary information

Supplementary Information.
