# Supplementary material for: Boron improves cardiac contractility and fibrotic remodeling following myocardial infarction injury
Source: Sci Rep. 2020 Oct 13;10:17138. doi: 10.1038/s41598-020-73864-w (PMC7553911; doi:10.1038/s41598-020-73864-w)
Supplement: Supplementary file 1 — Supplementary Information. [file 41598_2020_73864_MOESM1_ESM.pdf]

SUPPLEMENTARY INFORORMATION

Boron improves cardiac contractility and fibrotic remodeling following myocardial infarction injury

Rihab Bouchareb, Michael Katz, Najla Saadallah, Yassine Sassi, Shakir Ali, Djamel Lebeche

Supplementary Table 1: **Baseline echocardiography of rats before surgery (day 0).**

	Saline (n=7)	Boron (n=7)	p
**BW (g)**	350.08±20.38	348.02±18.23	0.54
**IVSd (mm)**	2.17 ± 0.61	2.09 ± 0.42	0.12
**LVIDd (mm)**	5.62±0.85	5.58±0.62	0.68
**LVPWd (mm)**	2.68±0.44	2.60±0.34	0.63
**IVSs (mm)**	3.11 ± 0.71	3.24 ± 0.65	0.89
**LVIDs (mm)**	2.6+ 0.52	2.55+ 0.35	0.87
**LVPWs (mm)**	3.35±0.23	3.41±0.19	0.45
**EF(Teich) %**	89.92±6.73	87.92±4.23	0.32
**FS %**	54.38±5.55	53.85±4.05	0.64
**E' (mm)**	4.27±0.36	4.30±0.25	0.49
**E (mm)**	92.11±20.41	90.21±19.12	0.29
**A (mm)**	60.14±15.23	62.03±10.63	0.56
**E/E'**	20.54±2.32	20.80±1.85	0.84
**E/A**	1.53±0.36	1.48±0.29	0.76

BW, body weight; IVSd, Interventricular septum thickness at end-diastole; LVIDd, Left ventricular internal dimension at end-diastole; LVPWd, Left ventricular posterior wall thickness at end-diastole; IVSs, Interventricular septum thickness at end-systole; LVIDs, Left ventricular internal dimension at end-systole; LVPWs, Left ventricular posterior wall thickness at end-systole; EF, ejection fraction; FS, Fractional shortening; TAPSE Tricuspid annular plane systolic excursion; E wave, mitral inflow peak velocity in early diastole; A, peak velocity flow in late diastole; E/A ratio is the ratio of the early (E) to late (A) ventricular filling velocities; E’, early relaxation velocity on tissue; E/E’ ratio of transmitral flow to mitral annular velocity. Data shown as mean±SD

Supplementary figures: Images of Original Western Blots

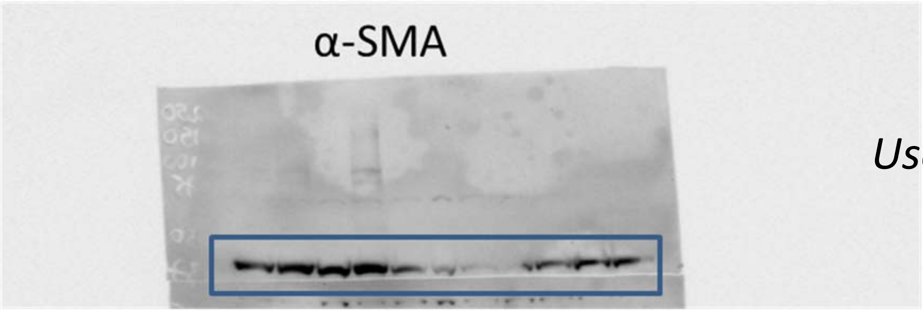

Used in Figure 4B

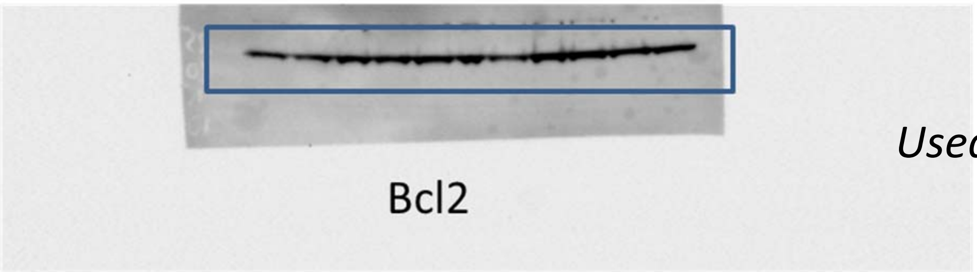

Used in Figure 5B

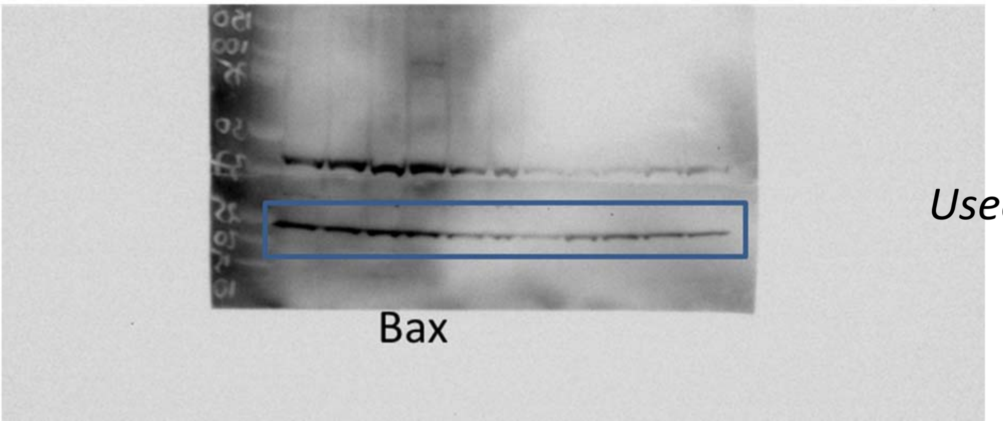

Used in Figure 5B

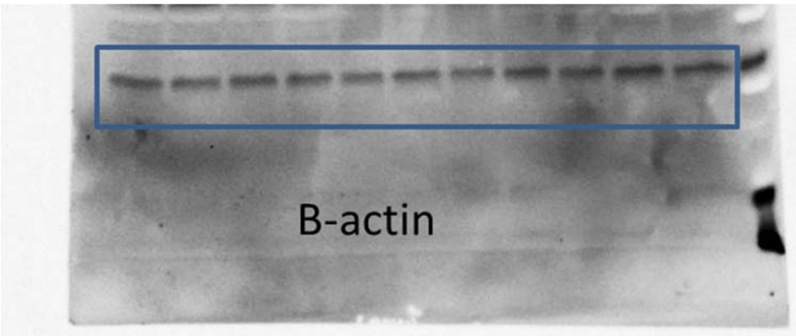

Used in Figure 4B and 5B
